# EVIDENCE-BASED FALL PREVENTION TRAINING FOR OLDER ADULTS IN RURAL COMMUNITIES

**DOI:** 10.1093/geroni/igae098.0064

**Published:** 2024-12-31

**Authors:** Sara Murphy, Andrew Crocker, Susanna Luk-Jones, Bergen Lemack

**Affiliations:** University of North Texas Health Science Center, Fort Worth, Texas, United States; Texas A&M AgriLife Extension Service, Amarillo, Texas, United States; University of North Texas Health Science Center at Fort Worth, Fort Worth, Texas, United States; University of North Texas Health Science Center, Fort Worth, Texas, United States

## Abstract

Falls among older adults is a public health issue affecting one-fourth of adults aged 65 and older annually and is a leading cause of unintentional injury death. Nearly 22% of older Texans live in one of the 199 rural counties, many without easy healthcare access. Texas has 71 counties with no hospital access, and only 83 rural counties have hospitals that provide 24/7 emergency care services. In response to these challenges, the University of North Texas Health Science Center Geriatric Workforce Enhancement Program (GWEP) partnered with Texas A&M Agrilife Extension Services (AgriLife) to expand A Matter of Balance (AMOB) fall prevention program for older adults in rural communities throughout Texas. Delivered as eight sessions, AMOB is designed to improve participant confidence, physical activity, and awareness through group activities in exercise, safety, assertiveness training, and problem-solving. Pre and post-surveys were administered to rate participant knowledge and confidence in managing fall-related situations on a scale from 1 (not sure at all) to 4 (absolutely sure). In 2023, AMOB sessions were held in 11 rural communities. Participants (n=182) were predominantly female (90.1%) aged 35 to 96 (average age: 75.88). A paired t-test comparing pre- and post-intervention scores revealed significant enhancements in knowledge about fall prevention strategies, improved ability to protect themselves during falls, enhanced physical strength, steadier balance, and better fall recovery skills. Evidence-based programs, like AMOB can be broadly disseminated to address challenges of falls among older adults at the individual and community level.

